# InterTADs: integration of multi-omics data on topologically associated domains, application to chronic lymphocytic leukemia

**DOI:** 10.1093/nargab/lqab121

**Published:** 2022-01-14

**Authors:** Maria Tsagiopoulou, Nikolaos Pechlivanis, Maria Christina Maniou, Fotis Psomopoulos

## Abstract

The integration of multi-omics data can greatly facilitate the advancement of research in Life Sciences by highlighting new interactions. However, there is currently no widespread procedure for meaningful multi-omics data integration. Here, we present a robust framework, called InterTADs, for integrating multi-omics data derived from the same sample, and considering the chromatin configuration of the genome, i.e. the topologically associating domains (TADs). Following the integration process, statistical analysis highlights the differences between the groups of interest (normal versus cancer cells) relating to (i) independent and (ii) integrated events through TADs. Finally, enrichment analysis using KEGG database, Gene Ontology and transcription factor binding sites and visualization approaches are available. We applied InterTADs to multi-omics datasets from 135 patients with chronic lymphocytic leukemia (CLL) and found that the integration through TADs resulted in a dramatic reduction of heterogeneity compared to individual events. Significant differences for individual events and on TADs level were identified between patients differing in the somatic hypermutation status of the clonotypic immunoglobulin genes, the core biological stratifier in CLL, attesting to the biomedical relevance of InterTADs. In conclusion, our approach suggests a new perspective towards analyzing multi-omics data, by offering reasonable execution time, biological benchmarking and potentially contributing to pattern discovery through TADs.

## INTRODUCTION

The study of the molecular mechanisms that lead to cancer was revolutionized by the advent of Next Generation Sequencing (NGS) (1,2). NGS extends from studies of whole genomes (whole-genome sequencing), to smaller regions of the genome (exome sequencing), the transcriptome (RNA-seq), the DNA methylome (bisulfite-seq) and the mapping of protein–DNA binding sites (ChIP-seq) (3). Using NGS to sequence the entire human genome can produce >100GB of raw data (4), thus leading to a whole new cadre of analytical challenges. From a computational perspective, the raw NGS-data are analyzed by established and widely accepted bioinformatics tools (e.g. bwa, TrimGalore, HISAT2, MACS2, R) (5), usually leading to a tabular representation of the captured information, with the different cases listed as columns and the locations on the genome in which the examined event occurred (e.g. mutation, gene expression etc.) as rows.

The integration of several types of data that originate from the same physical source (e.g. patient) yet investigate different ‘layers’ of cellular biology (e.g. the genome or the transcriptome, to name but two) remains a promising field, since there are no widely accepted methods to this end. The most common approaches for integrating different omics data tend to fall under two main categories: (i) comparing the gene list produced at the end of each individual analysis, with the working assumption that overlapping genes were influenced by mechanisms and processes operating in distinct ‘layers’ (6,7) or (ii) checking the correlation of two events that are associated with the same gene, using statistical methods such as spearman or Pearson correlation test (8,9), in order to infer the presence of a common mechanism. However, as interactions in biological systems are generally nonlinear, methods such as Singular Value Decomposition (SVD), Bayesian or non-Bayesian network-based were applied as extended data integration approaches (10). Although these methods are promising, they show instability and tend to over-fit the given dataset. Moreover, there are several existing tools that integrate different kinds of omics data but constrain the analysis only at the gene level (e.g. CNAmet, iGC, PLRS, Oncodrive-CIS), or focus on sample classification based on the driving clinical perspective (e.g. iClusterPlus and mixOmics) (10,11). Moreover, many existing tools consider pathway databases as a potential source for the extraction of a biological meaning across multiple independent omics datasets (12,13).

Going to a level of organization further than the simple chromosomal position, the introduction of NGS methods, like Hi-C, provides insight into chromatin organization such as the topologically associated domains (TADs). TADs represent segments of chromatin domains that are conserved in mammals (14,15) and are characterized by frequent interactions within themselves. Since the human genome is organized across all three dimensions in space, with gene regulation being driven also by the local folding of the chromosomes, multi-omics data integration requires information about the 3D chromatin structure. Moreover, recent studies have shown that integrating multi-omics data that also include TAD information, can offer novel insights into the regulation of genes implicated in tumor development (16–18). However, there is no complete framework published except from CESAM (17) which associates somatic copy-number alterations breakpoints with expression levels.

We developed an R-based framework, called InterTADs, for end-to-end analysis integrating multi-omics data while taking into account the 3D organization of the genome. The first step of the tool is the integration of the tabular output of multiple different types of NGS workflows (such as tables with expression values, mutations and DNA methylation values) into a single file. The tool then combines the joined representation of the multiple experiments, with the 3D organization of the genome, the TADs. It is important to highlight that the tool itself supports any type of genome segmentation, however, we consider that TADs (when available) can offer more insights towards the study of the effect, following the modern literature of 3D organization (19). Statistical analysis is performed according to predefined groups of interest (e.g. normal cells vs cancer cells), and the events related to multi-omics data (CpG site—CpGs, transcript, mutation, histone marker, etc.) which are consequently divided into the associated TADs based on the overlap of the chromosomal locations. Finally, enrichment analysis using KEGG database (20,60–62), gene ontology (GO) (21,59) and transcription factor binding sites (TFBS), as well as the relevant visualization options, are available for the statistically significant results. Our approach was tested on different omics datasets from 135 patients with chronic lymphocytic leukemia (CLL) (22), publicly available and retrieved from the Primary Cancer Cell Encyclopedia (PaCE) database.

CLL is a malignancy of mature B cell, the most common adult leukemia in Western countries (23), characterized by clinical and biological heterogeneity (24–26). Although the precise implicated mechanisms remain to be elucidated, the consensus is that CLL development and progression reflects an interplay between external (microenvironmental) drive, genetics and epigenetics (27,28). Two major molecular subtypes of CLL are recognized based on the molecular configuration of the B cell receptor immunoglobulin (BcR IG), more particularly the somatic hypermutation (SHM) status of the IGHV genes: cases with no or minimal SHM (‘IG-unmutated’ CLL, U-CLL) follow considerably more aggressive clinical courses compared to those with a significant SHM load (‘IG-mutated’ CLL, M-CLL) (29–31). Regarding genetics, recurrent gene mutations and chromosomal abnormalities are detected in the great majority of CLL patients with subgroups of patients displaying different landscapes of genomic aberrations exhibiting distinct clinical behavior and response to treatment (32–34). Concerning the former, mutations in the *TP53, NOTCH1, SF3B1, ATM* and *BIRC3* genes occur at a frequency of ∼2–10% in general cohorts of untreated patients, whereas their frequency increases among patients with progressive or high-risk disease (33–35). Concerning the latter, the most common cytogenetic abnormalities, ranking from high to low risk, are: del(17p), del(11q), trisomy 12, del(13q) (26). Furthermore, differential DNA methylation and histone modification profiles have been reported for different CLL prognostic subgroups, for example cases with U-CLL versus M-CLL genes and the presence or absence of trisomy 12 (8,22,36–38).

Due to its great heterogeneity and well characterized prognostic/predictive biomarkers, CLL provides a paradigmatic case to decode complex associations of the events within the same TADs. We report the high biological benchmarking of the InterTADs method, since the produced results clearly reflect the existing literature, such as the significance of SHM status. Moreover, we highlight the reduction of the heterogeneity by integrating the data on TADs compared to individual events. Finally, we show that InterTADs provides an efficient means to decode complex associations between omics data within TADs, assisting in the discovery of molecular pathways and transcription factors (TFs) relevant for disease pathogenesis.

## MATERIALS AND METHODS

### Overview

**Figure 1.**
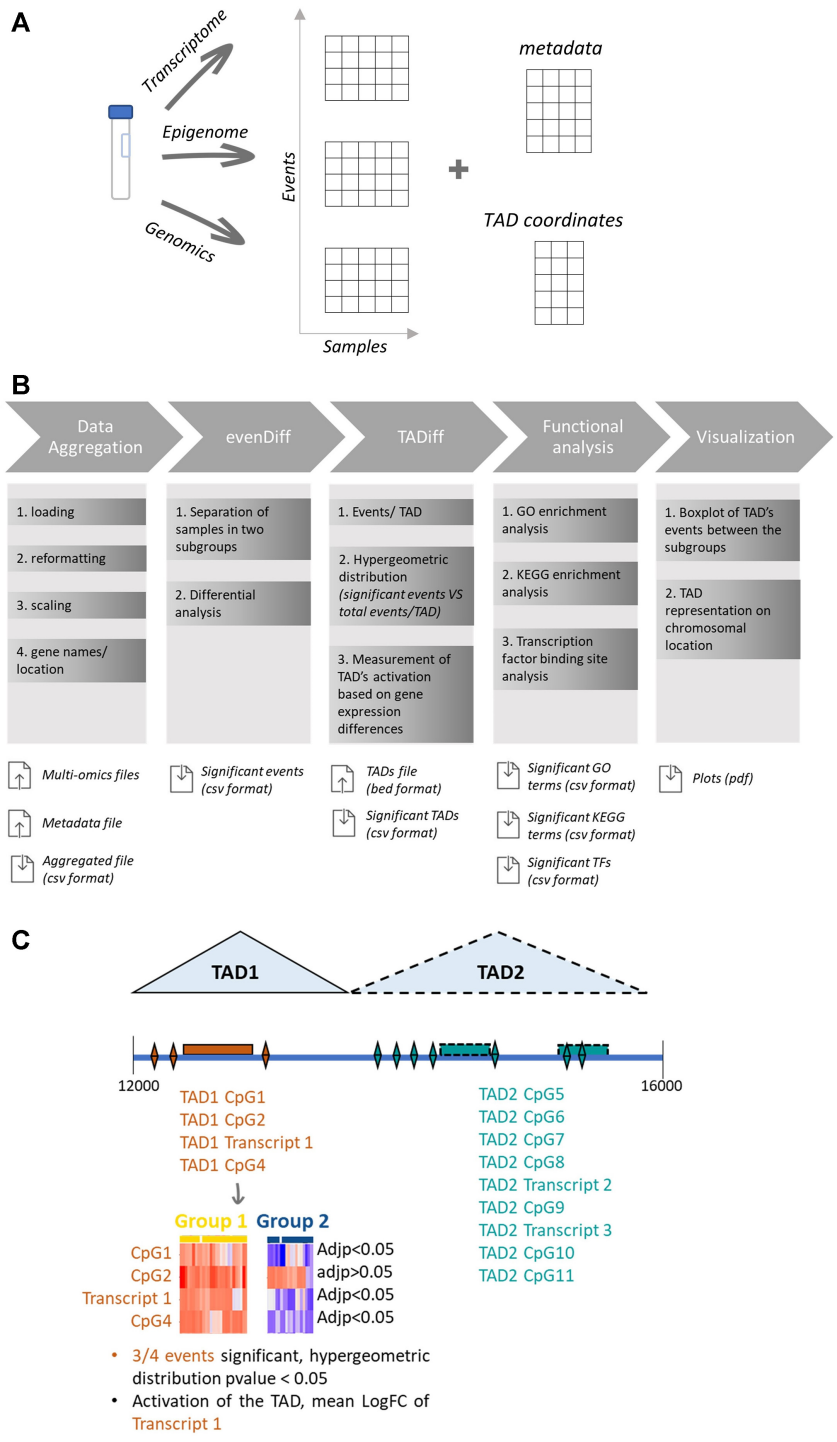
(**A**) Schematic diagram of input files for InterTADs. (**B**) Schematic diagram of InterTADs. Input multi-omics data are loading, reformatting, scaling and annotating to the genome in order to generate a single aggregated file. The events of the aggregated file are mapped on the TADs and statistics are provided for single events and for the integrated table through TADs. Finally, functional analysis and plotting functions are available. (**C**) Schematic diagram of the data integration regarding TADs. The upper part shows the grouping of the event in each TAD and then with a special focus on TAD1 an explanation of the criteria included in the TADiff module.

Briefly, the data aggregation module contains functions for loading, reformatting and scaling of the input files, and ultimately constructs a single table. Subsequently, each event of the integrated table is characterized according to the related gene and the genomic features (exon, intron etc.). Regarding the 3D organization, all events are grouped into corresponding TADs based on the overlap of the chromosomal regions. A statistical analysis is then performed, which includes the evaluation of the differences of the (i) events and (ii) TADs between the predefined groups of interest (e.g. normal cells versus cancer cells), retrieved by a user-provided *meta-data* file. As an additional post-processing step, enrichment analysis using KEGG database, GO and TFBS options are available for the downstream analysis. Finally, visualization scripts produce plots of the events on the chromosomal location of a TAD and dot plots based on the values of the events on a TAD, considering the predefined groups for both options. It is worth noting that the visualization outputs include figures related to the enrichment analysis using KEGG database, GO and TFBS highlighting the significant terms on bar plots. Our approach (Figure 1) can be applied to any kind of NGS or array-based experiment, and any cohort size and integrates with the TAD boundaries using either publicly available Hi-C data or any custom-defined segmentation of the reference genome used in the analysis.

### Workflow

We split the InterTADs workflow into four main phases: (i) automation of the multi-omics data aggregation, (ii) introduction of the biological knowledge regarding the 3D organization of the genome through the TADs, (iii) functional analysis of the significant results and (iv) visualization of the statistically significant results (Figure 1):

- **Data aggregation:** The first phase includes the automated process of reading and formatting all inputs into a single file. Since the tool is focused on multi omics data, the samples with missing data will be automatically excluded from the analysis. Omitting this, the user can apply two different scenarios including (i) the minimum information that is present in most of the cases (ii) the higher number of multi-omics layers even if it is not present in all samples. The input tab-delimited files contain the coordinates of each event (CpG, transcript, mutation etc.), and the corresponding score values. These files are produced by tools performing the analysis of the raw data such as HISAT2 (39), featureCounts, MACS2 (40), minfi (41), GATK (42). Along with these files, a *meta-data* file is created containing information about the mapping between the files’ columns. The output of this step is a tab-delimited table in which the first column corresponds to the chromosome that the specific observation (table row) belongs to. The second and third columns contain information about the TAD name and the TAD’s start and end positions while the fifth to seventh columns contain information about the event's ID (CpG, transcript, mutation etc.) and chromosomal location (start and end positions). The next two columns (#8 and #9) store information about associated genes (Gene ID and Gene functionality) while the tenth column points to the input an event comes from. Finally the rest of the columns correspond to the samples’ count or freq values. The Data aggregation phase consists of five steps (Figure 1):
  1. **Loading**: First, all inputs are read and loaded regardless of the source of each individual file.
  2. **Reformatting**: Next, each file is transformed into a data table based on the given *meta-data* file. This transformation ensures that the same index columns from different tables point to the same physical source (chromosome information, patient ID etc.).
  3. **Scaling**: In order for any further analysis to be possible, all tables are transformed so that they correspond to the same scale. A range between [0, 100] has been chosen for convenience purposes. Hence, numeric data, which contain frequency score values in the above range, are slightly (DNA methylation) or not at all changed (mutation data). On the other hand, a function is applied to count values so that they correspond to the desired range. The transformation process is as follows; supposing that *E* corresponds to expression counts, then a logarithmic scale is applied:
    \begin{eqnarray*}{\rm{\ }}{E_{log}} = \ {\rm{ln}}\left( E \right)\end{eqnarray*}
    Later on, a vector with all maximum values of the columns of ${E_{log}}$ is created:
    \begin{eqnarray*} {{\boldsymbol{E}}_{{\boldsymbol{max}}}} = \mathop {\max }\limits_j {E_{log}},\ {\rm{where\ refers\ to\ column\ index}} \end{eqnarray*}
    and a new matrix is generated by calculating the ratio between the maximum values and the desired range:
    \begin{eqnarray*} {E_{new}} = {E_{log}}\ \cdot 100/{{\boldsymbol{E}}_{{\boldsymbol{max}}}} \end{eqnarray*}
  4. **Gene names/location:** Moreover, for every event on the new integrated matrix, the gene names and locations (exon, intron, cds etc.) are retrieved based on the chromosomal location of the event. This module includes options for either hg19 or hg38 annotation according to the reference genome of the multi omics data.
  5. **TAD annotation:** Finally, a BED file containing information of segments of the genome, such as TADs (segments), is provided and compared with each event for overlaps between their chromosomal coordinates. The TADs are conserved sites for a specific cell type and there are several publicly available files on UCSC, on the ENCODE project and also Hi-C experiments on GEO DataSets. Additionally, InterTADs can perform with a user-specified bed file containing segments of the users’ choice instead of the TADs.
- **Reverse methylation values:** The output file of the Data Aggregation phase can be used as input to the prepare_methylation_values module. The script provides functionality for filtering specific locations of methylation values (e.g. promoter, intergenic) and reversing them so that low/high methylation frequencies correspond to high/low values.
- **EvenDIff:** The output file of the Data Aggregation phase is the input for the EvenDiff module. At this phase, the samples are split in two subgroups according to a predefined metadata file that includes a list of sample IDs and the corresponding group, e.g. normal/tumor. Then, statistical analysis between the two subgroups is performed based on the limma package (43). The output of this module includes the statistically different events between the predefined groups, together with the output of the limma package (logFC,AveExpr, adj.P.Val, etc), chromosomal location and the individual values of each sample.
- **TADiff:** The output file of the Data Aggregation phase is also provided as input for the TADiff module. The tool splits the samples in two subgroups, according to a predefined metadata file, e.g. normal/tumor. The statistical analysis between the two subgroups includes:
  - Filtering of the statistically significant events from the evenDiff divided across the TADs.
  - Hypergeometric distribution test using the number of the significant events of the TAD compares it with the total number of events in the TAD.
  - Measurement the activation of the TADs through the phenotypic outcome (i.e. gene expression) calculating the mean of absolute numbers of logFC.

The output of this module is a table with the statistically significant TADs defined by a high number of differential events between the groups and a phenotypic outcome in gene expression (i.e. differentially expressed genes between the groups). Also, it includes the statistical analysis of this phase in each TAD and their associated events with their values in each sample.

- **Functional analysis:** The next phase of the workflow includes an enrichment analysis with GO Biological Process and Molecular Function Terms, KEGG pathways and TFBS. The output files of the EvenDiff and TADiff phases are the main inputs of this phase, as the analysis is performed on the events confirmed as significant by the previous steps. It consists of two parts:
  - GO/KEGG enrichment using the Enrichr tool (44,63,64) and the annotated gene names of the events.
  - Motif enrichment using the allele sequences of the events and specifically for the expression events, the promoter regions, provided as inputs to the PWMEnrich tool (65). The Ensembl Rest API (45) is used to extract representation of the respective sequences at the nucleotide level from their corresponding chromosomal coordinates.
  - The enriched terms are matched with the corresponding TADs of the events they were found into. Then, a hypergeometric distribution test is performed using the number of genes matched to each term per TAD and the total number of genes annotated to the term to determine the significance of each TAD per enriched term.
- **Visualization:** The visualization phase includes three options: (i) dot plots for the two subgroups based on the values of the events of each TAD, (ii) dot plots for the two subgroups based on the mean values of the cases of each event and (iii) chromosomal representation of TADs. In more detail, the dot plot takes into account the associated events of a TAD of interest and plots the values of the cases between the two subgroups. The second option is a dot plot based on the mean of each event in each subgroup accompanied by a connecting line. Also, a violin plot is generated on the same plot showing the distribution of the mean values. The third option takes as input the integrated matrix and a desired chromosomal location, and produces plots showing the chromosomal location of the TAD of interest on the x axis and the associated events combined with their values on the y axis. The plots are generated based on each case separately or on each group. Also, a single plot with the differences of the events between the groups is produced. Additionally, regarding the functional analysis there is a visualization output of the significant terms in each module (analysis using KEGG database, GO and TFBS) using bar plots.

Considering all the steps, the InterTADs generates different output files:

- *integrated-table.csv*: A table contains all the events of the input omics data included the ID of the event (e.g. cg02913364, chr1 100503564:T:C etc.), the chromosomal location (e.g. chromosome, start, end), gene names, gene locations (transcript, exon, threeUTR)
- *integrated-tad-table.csv*: The *integrated-table* containing additional information about the TAD in which each event belongs to.
- *integrated-tad-table-methNorm.txt*: The integrated-tad-table after reversing methylation values as described in Materials and Methods section.
- *summary.txt*: A mapping file showing what kind of information (methylation, gene expression etc.) is coming from each source.
- *IGHV_evenDiff.txt*: A tab-delimited table containing the results for the statistical analysis of the evenDiff module on the IGHV meta-data column.
- Summary_evenDiff.txt: A summary table with information of the evenDiff statistical analysis.
- *IGHV_TADiff.txt*: A tab-delimited table containing the results for the statistical analysis of the TADiff module on the IGHV meta-data column.
- Summary_TADiff.txt: A summary table with information of the TADiff statistical analysis.
- *over-represented enriched terms.csv*: A table of the enriched terms and their corresponding p-values and adjusted p-values, grouped with regard to the TAD they were found into.
- *enriched terms in different TADs.csv*: A table containing the information as the *over-represented enriched terms.csv* file, grouped with regard to the enriched term.
- *prepared sequences info.csv*: A table containing the information (e.g. chromosome name, start, end) of the merged sequences after the extension of the methylation events, as well as the IDs of the initial events.
- *seq_perTADs.fasta*: A file with the allele sequences produced using the Ensembl Rest API.
- *report MotifEA.txt*: A file containing the output tables of the PWMEnrich tool for each TAD.

The *over-represented enriched terms.csv* and *enriched terms in different TADs.csv* files are produced independently for each kind of term the data are enriched with; GO Biological Process (BP) terms, GO Molecular Function (MF) terms, KEGG pathways and TFs.

### Data

The proposed method was evaluated using data from a large CLL series retrieved from the Primary Cancer Cell Encyclopedia (PaCE) database, directly as data tables. The detected mutations of the study were used as additional information to the metadata since there are targeted sequencing results. Finally, we used the matrix of DNA methylation values (based on Illumina 450K BeadChip Arrays), the matrix with expression values (based on RNA-seq) as the input multi-omics file for our tool. Finally, TADs from the cell line GM12878 were used (36) which is a lymphoblastoid B-cell line widely used to characterize the epigenome in CLL (8,18,36,37). Additionally, and in order to assess the impact of using well-defined TADs versus randomized segments of DNA sequence, we generated five different BED files containing randomly generated segments of similar size distribution with the original TAD file, and ran the entire pipeline with the same parameters (results shown in Supplementary Figures S1–S3). The two omics datasets contained 221 565 rows with DNA methylation events (CpGs) and 47 639 rows with gene expression events (transcripts). For the whole analysis, only events on chromosomes 1–22 were included. We generated the metadata file by filtering out the makers whose presence was in less than five patients.

### Implementation

The tool is implemented as a standalone R script. The input multi-omics files are BED-formatted containing the coordinates of each event (mutation, CpG site, transcript etc), and the corresponding score values. In more detail, the input files have to include in the first column a unique identifier (e.g. cg00000029, XLOC_032721, mut_1, etc.) for each event, in the next second to fourth columns the BED format information (i.e. chromosome, start, end), and in the rest of the columns the values for each patient. These files are produced by tools performing the analysis of the raw data such as HISAT2 (39), featureCounts, MACS2 (40), minfi (41), GATK (42), etc. In our study case, the format of the omics data were transformed to a BED-format adding the scores of each patient using the library IlluminaHumanMethylation450kanno.ilmn12.hg19 in R for the CpG events; and, the GTF file from StringTie of the HISAT2 pipeline. In order for the algorithm to run properly, all files are placed into two folders, named *freq* and *counts*, based on the type of information they are carrying (frequency score values or count values). Along with these files, a *meta-data* file is created containing information about the mapping between the files’ columns. The overlap of the ranges between the events and the TADs is tested using the R package GenomicRanges (46). The plotting functions were generated using ggplot2, gghalves and karyoploteR (47).

## RESULTS

### Unsupervised analysis using evenDiff and TADiff module of InterTADs leads to a biologically relevant clustering of cases

InterTADs was applied on previously published omics data from 135 CLL as described in the Data Section above. The aggregated table, as constructed after application of the evenDiff step, contains 267 650 events, i.e. CpG sites, transcripts. These events were found on 3034/3036 (99.8% 0 TADs in the GM12878 cell line, after the TADiff step.

**Figure 2.**
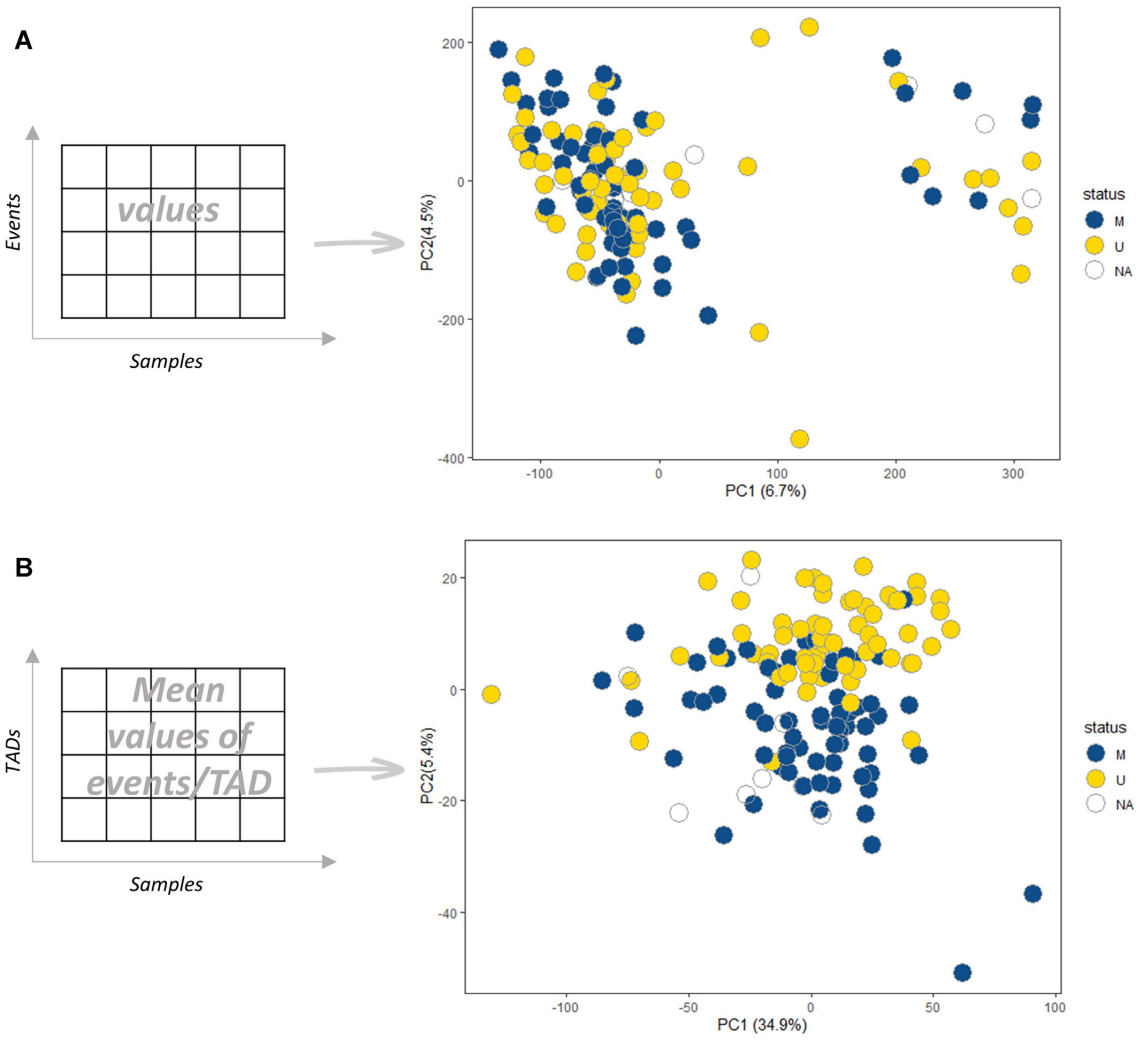
Principal component analysis showing components 1 and 2 in subgroups regarding the IG SHM status based on A. the aggregated table of evenDiff module and B. the integrated table through TADs of the TADiff module calculated the mean of the events for each TAD.

To demonstrate the ability of the tool in revealing the most significant results compared to the individual events, we performed unsupervised principal component analysis on both the aggregated data table and the TAD-associated table. The analysis was performed according to the predefined metadata file, in which the samples are characterized based on the group of interest. We selected the SHM status of IG genes since it is perhaps the key biological statifier in CLL and, moreover, remains stable overtime, hence contrasting genomic aberrations which tend to change with disease evolution. The aggregated table shows a high heterogeneity and low explained variance of the Principal Components (PCs) (Figure 2A). Aggregating the integrated events across the related TADs and calculating the mean of the events for each TAD, a clear separation emerges between M-CLL and U-CLL (Figure 2B), highlighting the potential of InterTADs to provide biologically meaningful results. Also, we noticed that the explained variance of the PCs is increased after TADiff. Due to the fact of the overpressenation of CpGs compared to transcript on the TAD matrix, we performed unsupervised hierarchical clustering analysis taking into account (i) the CpGs and the transcripts (Supplementary Figure S4A) and (ii) only the CpGs (Supplementary Figure S4B). The results show different clustering between the two approaches and the integration of two multi-omics layers concluded to a better clustering of the SHM status.

### Exploratory analysis of the evenDiff module supports the relevance of the InterTADs tool

**Figure 3.**
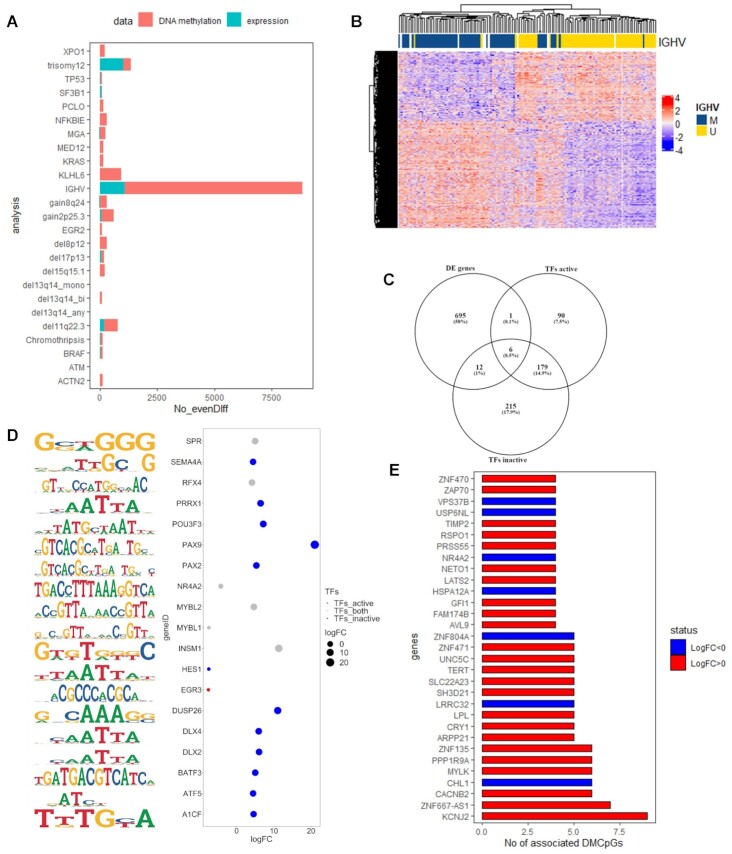
(**A**) Bar plot showing the number of the statistically significant events (x-axis, No-evenDiff) in each subgroup of the metadata. Changes on CpG level is highlighted with orange and on gene level with blue. (**B**) Hierarchical cluster analysis of the 8859 events from the M-CLL/U-CLL comparison. (**C**) Venn diagram showing the overlap of the TFs which showed enrichment on active and inactive region between M-CLL and U-CLL with the differentially expressed genes between these groups. (**D**) 19 TFs which showed statistically significant enrichment based on the 8859 events and statistically significant differences in the expression levels between M-CLL and U-CLL. Blue circles highlight the enriched TFs on inactive regions, red circles highlight the enriched TFs on active regions and gray the TFs which showed enrichment on both regions. The left panel shows the motifs for each TF. (**E**) Bar plot showing 31 genes which were targeted with more than three CpGs. Blue corresponds to down-regulated genes and red to up-regulated genes in expression analysis.

Using the aggregated table, we investigated the significant events that were identified in several subgroups of CLL, such as. cases which carried del(11q), trisomy 12, *TP53* gene aberrations [i.e. del(17p) and/or *TP53* gene mutations], or M-CLL/U-CLL status, by applying the evenDiff module. We found significant differences (adj-pvalue < 0.01) regarding U-CLL/M-CLL (*n* = 8859 events) and trisomy 12 (*n* = 1341) (Figure 3A, Supplemental Tables S1 and S2). Hierarchical clustering analysis of 8859 events revealed distinct patterns between M-CLL versus U-CLL (Figure 3B). Then, by applying the functional analysis module on the significant events table of the M-CLL/U-CLL categories, we found statistically significant results (adj-*P*-value < 0.05) in KEGG pathways for inactive events/related genes in U-CLL versus M-CLL which were enriched in Allograft rejection, Graft-versus-host disease, Type I diabetes mellitus, and Cytokine-cytokine receptor interaction. Moreover, the TFBS analysis revealed significant results for both active and inactive events relevant to B cell/CLL biology. Moreover, by focusing the analysis only on the TFs showing differences on the expression levels of U-CLL and M-CLL as well, we ended up with 19 key TFs (Figure 3C and D). Finally, analyzing the aggregated table, 31 genes were targeted with more than three events (Figure 3E) such as *KCNJ2, CRY1, ZNF667-AS1, CACNB2, CHL1, MYLK, PPP1R9A, ZNF135, ZAP70*.

### Exploratory analysis of TADiff module revealed differential TAD activation in M-CLL versus U-CLL

Applying the TADiff module, we explored the significant TADs in different subgroups of CLL based on the corresponding metadata. We applied thresholds, as descripted in methods section, of:

- differential analysis of the events ( $| {logFC} | = {\rm{\ }}2,{\rm{\ }}adj{p_{value{\rm{\ }}}} \lt 0.01)$)
- hypergeometric distribution test (${p_{value}} \lt 0.01$)
- Measurement the activation of the TADs through the phenotypic outcome ($mea{n_{logFC}} 2$)

**Figure 4.**
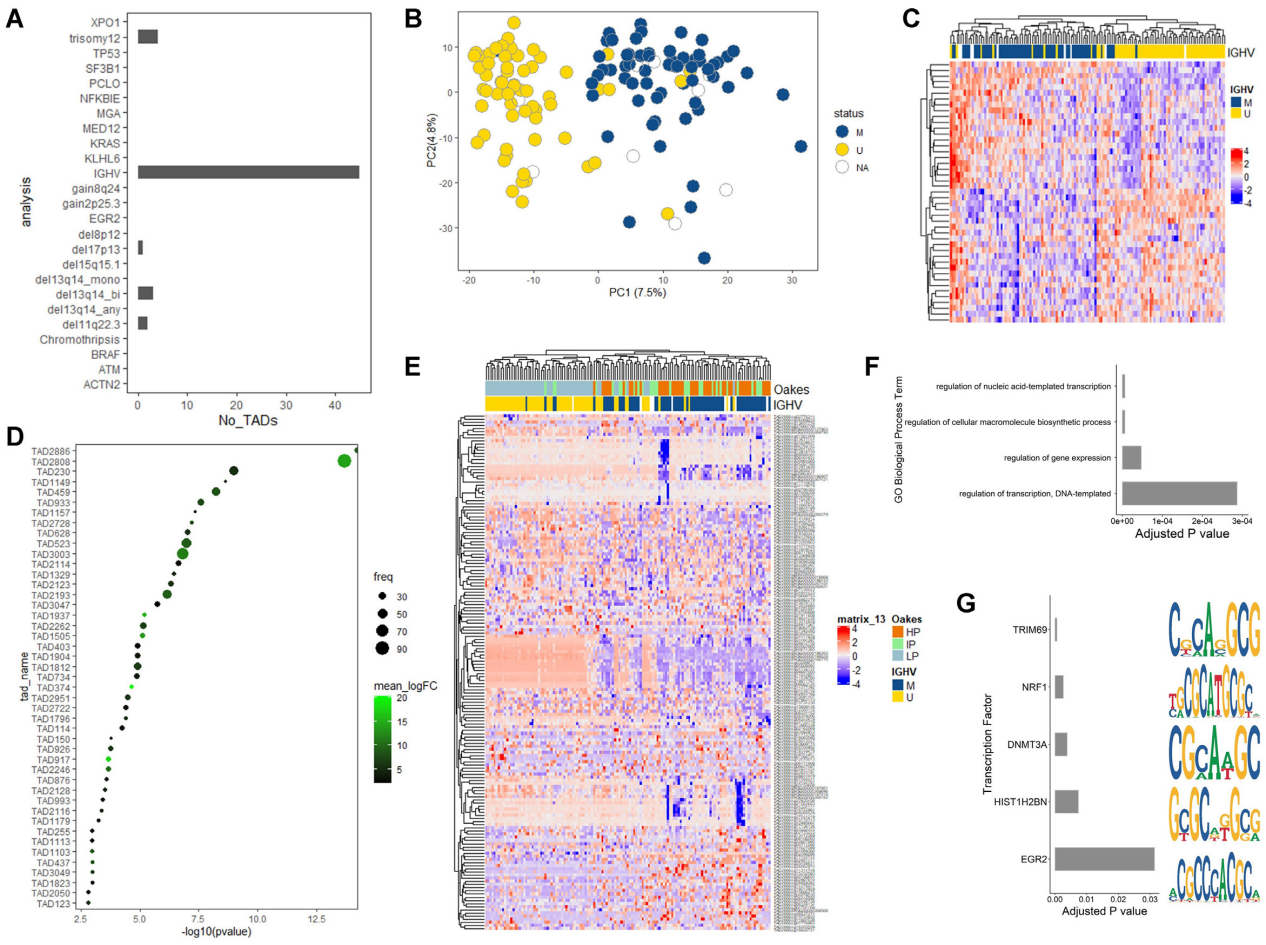
(**A**) Bar plot showing the number of the statistically significant TADs (x-axis, No_TADs) in each subgroup of the metadata. (**B**) Principal component analysis showing components 1 and 2 in the IGHV subgroups based on 2,251 events of 45 TADs. (**C**) Hierarchical cluster analysis of the 45 TADs from the M-CLL/U-CLL comparison calculating the mean of the absolute differences of the events in each TAD. (**D**) Dot plot showing the statistically significant TADs of the M-CLL/U-CLL comparison on y-axis and the −log_10_*P-*value of the hypergeometric distribution test. The color code shows the activation of the TADs through the phenotypic outcome (mean of logFC based on gene expression levels) and the size of the dots represents the frequency of the statistically significant events in each TAD compared the total number of events of the TAD. (**E**) Hierarchical cluster analysis of TAD2886 including 164 events (147 CpGs, 17 transcripts). (**F**) Bar plot showing the significant GO terms based on the 37/160 events of TAD2886 on y-axis and the adj-pvalue of the GO enrichment analysis in x-axis. (**G**) Bar plot representing the statistically enriched TFs based on the 37/160 events of TAD2886 on y-axis and the adj-pvalue of the enrichment analysis in x-axis. The right panel shows the motifs for each TF.

The results showed 45 statistically significant TADs between M-CLL and U-CLL (Figure 4A, Supplemental Table S3). The 2251 events of 45 TADs were used on PCA and showed distinct separation of M-CLL from U-CLL (Figure 4B). Next, we calculated the mean of the absolute differences of the events in each TAD and performed hierarchical clustering analysis, which revealed different activation patterns between the two groups (Figure 4C).

The most significant TAD, based on the criterion of the p-value of the hypergeometric distribution, was TAD2886 (Figure 4D) at chr19, 56 900 000–57 700 000 [*P*-value < 0.001, mean_logFC(activation) = 9.167059]. This TAD includes 164 events (147 CpGs, 17 transcripts) of which 37 were found to be statistically significant. Hierarchical clustering analysis, based on 164 events, revealed a clear separation between U-CLL and M-CLL (Figure 4E, Supplementary Figure S5) cases, therefore highlighting the appropriate selection of criteria in order to successfully detect 3D interactions. Additionally, the functional analysis based on the significant events (37/160) revealed significant GO terms (Figure 4F, Supplementary Figure S6), such as regulation of transcription, but also relevant TFs for the pathogenesis of the disease, such as EGR2, and regulators of chromatin, such as TRIM69, HIST1H2BN and DNMT3A (Figure 4G, Supplementary Figure S7).

### Benchmarking

**Figure 5.**
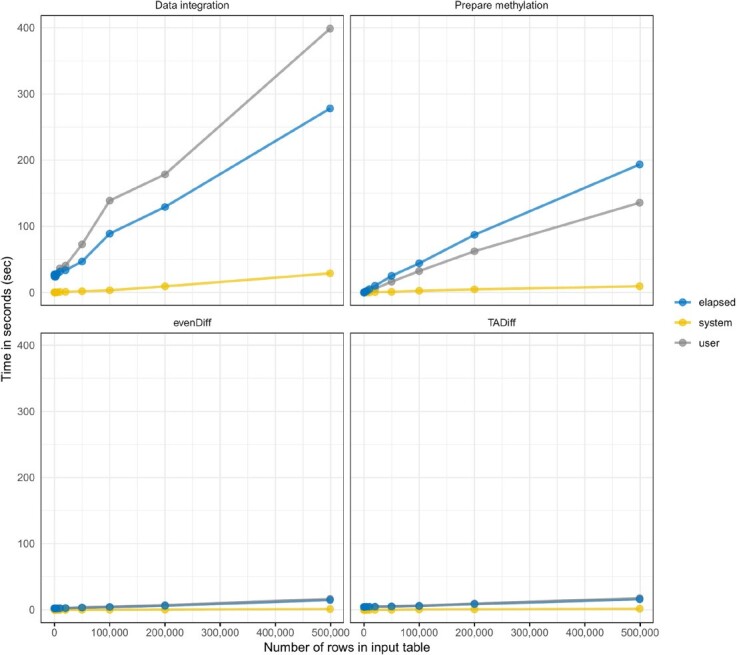
Compute times for each phase of InterTADs, showing time (in seconds) as a function of the number of rows of the artificial input table.

For the evaluation of the tool's computational time, twelve different subsets of the original datasets were produced containing 100, 200, 500, 1000, 2000, 5000, 10 000, 20 000, 50 000, 100 000, 200 000 and 498 832 number of rows (e.g. events; CpG, expression, mutation etc.). All experiments were executed on an SSD drive computer with 32GB RAM at 2.60 GHz and a 64-bit operating system. Figure 5 shows the compute times for each phase of InterTADs (e.g. Data integration, Prepare methylation values, evenDiff, TADiff) as a function of the produced datasets’ size. The functional analysis was excluded from the computational benchmarking as it is strongly affected by the response times of the data base that are used.

### Complexity

The complexity of the proposed framework is analyzed for every module below. For the analysis we assume that if each statement is ‘simple’ (only involves basic operations) then the time for each statement is constant and the total time is also constant:${\mathrm O}( 1 )$:

As described above, the first part of the multi-omics integration pipeline is the Data Integration phase. In order to analyse the complexity of this module the following constants are defined:

-
${N_{files}}$ : Number of files to read.
-
${N_{events}}$ : Number of total events retained
-
${N_{TADs}}$ : Number of TADS
-
${T_{read}}$ : response time for reading a file
-
${T_{filtering}}$ : response time for filtering events
-
${T_{GenomicOverlap}}$ : response time for finding genomic overlaps

Consequently, the complexity for each part of this script is:

- Reading the input files: $O( {{N_{files}} \cdot {T_{read}}} )$
- Filtering events: $O( {{N_{events}} \cdot {T_{filtering}}} )$
- Annotating with gene features (e.g. Gene ID, Gene location): $O( {{N_{events}} \cdot {T_{GenomicOverlap}}} )$
- Annotating with TADS: $O( {{N_{TADs}} \cdot {N_{events}} \cdot }$  ${T_{GenomicOverlap}} )$

The overall complexity of this module is estimated as follows:

\begin{eqnarray*}&&O({N_{files}} \cdot {T_{read}} + {N_{events}} \cdot ({T_{filtering}} \\ &&\quad+{T_{GenomicOverlap}} + {N_{TADs}} \cdot {T_{GenomicOverlap}}) )\end{eqnarray*}

For the second part of the pipeline, preparing methylation values module, *N_samples_* is defined as the number of total samples to be analyzed and the total complexity results in *O*(*N_samples_*).

The next two phases of the pipeline are the EvenDiff and the TADiff modules. For the EvenDiff part the following variables are defined:

- *N_meta–data_*: number of meta-data to be compared
- *T_limma_*: response time for *limma* differential analysis

The complexity results in *O*(*N_meta–data_*⋅*T_limma_*).

In addition, for the TADiff module the following parameters are defined:

- *N_meta–data_*: number of meta-data to be compared
- *T_limma_*: response time for *limma* differential analysis, which result in the complexity of *O*(*N_meta–data_*⋅*T_limma_*).

Finally, the theoretical complexity of the functional analysis phase, performed for one file, can be roughly calculated as follows. Firstly, the following constants are defined:

- *E*: number of events of the file
- *N_T_*: number of TADs corresponding to the events
- *N_e_*: number of enriched terms annotated to the events
- *T_Enrichr_*: response time of the Enrichr tool
- *T_PWMEnrich_*: response time of the PWMEnrich tool
- *T_Ensembl_*: response time of the Ensembl Rest API

Considering the workflow described in the Materials and Methods section, the complexity is estimated as follows:

- GO/KEGG enrichment part: *O*(*N_T_*〉*T_Enrichr_*)
- Motif enrichment part: *O*(*E*⋅(*T_PWMEnrich_*⋅*T_Ensembl_*)
- Analysis of each enriched term per TAD: *O*((*N_T_*⋅*N_e_*)^2^)

Assuming that the computational time of the TAD annotation and the hypergeometric distribution test is negligible compared to the response times of the web tools, an overall estimation of the theoretical complexity is:

\begin{eqnarray*}O\left( {{N_T} \cdot {T_{Enrichr}} + E \cdot \left( {{T_{PWMEnrich}} \cdot {T_{Ensembl}}} \right)} \right)\end{eqnarray*}

It is worth mentioning that in case the file was produced by the EvenDiff phase, the first term degenerates to *O*(*T_Enrichr_*).

## DISCUSSION

NGS technologies have impacted massively on the life sciences, especially in cancer research. Through global scientific communities and consortia, such as The Cancer Genome Atlas (TCGA) (48), the International Cancer Genome Consortium (ICGC) (49), BLUEPRINT (50) etc., high-quality data and corresponding metadata of over 20,000 tumor genomes are available worldwide.

Despite the increasing amount of data, however, there is no single approach to efficiently integrate multi-omics data that originate from the same source (e.g. patient). Here we propose a novel method, implemented as a tool named InterTADs, which provides a complete end-to-end framework for the analysis of multi-omics data, that are either available in-house or through public repositories. The implementation of InterTADs includes (i) generating a single file from multi-omics inputs, (ii) finding significant differences in the events and the TADs between predefined groups of interest, (iii) performing functional analysis based on GO, KEGG and TFBS analysis and (iv) visualizing the TADs of interest and significant terms of the functional enrichment analysis. Our approach clearly supports efficient pattern discovery in multi-omics data by decreasing the heterogeneity (and therefore potential noise) across higher level organizational units (i.e. TADs), as compared to the individual datasets/ events.

In regard to other existing tools (10), InterTADs falls under the genome-wide approaches category, by separating the genome into predefined segments, with TADs being the case in study. Applying this approach, we omit the gene level analysis and a more random windows analysis approach, which generates sliding windows within the chromosome by taking into account the chromatin configuration and the high level of interactions within the TADs. Also, the users could upload a bed file with segments of their choice instead of the TADs file. Our proposed tool is applicable to any kind of omics data.

By applying InterTADs on 135 CLL cases, we validate our results by reproducing the outputs from previous publications, but we also suggest a new pattern discovery approach through the data integration within the TADs. In fact, initial unsupervised principal component analysis (PCA) disclosed a distinct separation of M-CLL from U-CLL based on the integrated table through TADs (TADiff module) compared to individual events (evenDiff module) which represents a biological benchmarking of the tool considering the significance of IG SHM status as the key biological stratifier in CLL (29–31). Moreover, the explained variance of the PCs is increased after TADiff, highlighting the value of the tool as a pattern discovery approach.

In more detail, we identified clear and statistically significant differences between events by applying the evenDiff on the categories relating to IG SHM status and the presence of trisomy 12. Several publications have already highlighted the differential patterns on transcriptomics and epigenomics layers (8,19,33–35) in these disease subgroups, hence suggesting the biological relevance of InterTADs. Focusing on the IG SHM status, we found genes that were targeted by more than one event, such as *KCNJ2* (51)*, CRY1* (51)*, ZNF667-AS1* (52)*, MYLK* (51)*, ZAP70* (53), all of which have been previously identified in previous reports. We also observed that events of inactive regions in U-CLL were enriched for binding sites of several TFs relevant to B cell/CLL biology, while also showing differential expression between the two groups, including the ATF5 (54), MYBL2 (55). Applying the TADiff module, we revealed differentially active TADs in M-CLL and U-CLL cases. The hierarchical clustering analysis of TAD2886, which showed the lowest p-value (hypergeometric distribution), revealed a clear separation of M-CLL from U-CLL cases, including all associated events (both significant and non-significant). These results uncover 3D interactions inside the TADs and highlight the crucial role of specific TFs participating in this complex interplay such as the chromatin regulator *TRIM69, HIST1H2BN* and *DNMT3A*, as well as genes that play a role in gene regulation i.e. *members of ZNF protein family*.

InterTADs is an open-source R package, easily applicable to any type of omics data. The tool is in line with the Open Science and FAIR principles (Findable, Accessible, Interoperable, Reusable) for research Software (56), and is freely available on GitHub under an MIT license. Altogether, InterTADs aggregates different omics data and integrates them within TADs. The publicly available data of multi-omics and Hi-C experiments from primary cells are increasing (57,58) and future studies will uncover more relevant associations of the related events within compartments or TADs highlighting the importance of the InterTADs tool. The user can upload the TADs displaying differences between groups (e.g. U- versus M- CLL) captured by Hi-C and apply the tool on a supervised perspective for further pattern discovery. Our method offers a new perspective towards analyzing multi-omics data, by streamlining the entire process, and by incorporating a meaningful representation of information structure, biological benchmarking, pattern discovery and clear visualization options.

## DATA AVAILABILITY

InterTADs is an open-source tool implemented in R and licensed under the MIT License. The source code is freely available from https://bio.tools/InterTADs (GitHub repo https://github.com/BiodataAnalysisGroup/InterTADs). The InterTADs was applied on data from a large CLL series retrieved from from the R package BloodCancerMultiOmics2017 ((https://bioconductor.org/packages/release/data/experiment/html/BloodCancerMultiOmics2017.html) of the Primary Cancer Cell Encyclopedia (PaCE) database (http://pace.embl.de/), directly as data tables.

## SUPPLEMENTARY DATA

Supplementary Data are available at NARGAB Online.

## Supplementary Material

lqab121_Supplemental_Files
