# Supplementary material for: InterTADs: integration of multi-omics data on topologically associated domains, application to chronic lymphocytic leukemia
Source: NAR Genom Bioinform. 2022 Jan 14;4(1):lqab121. doi: 10.1093/nargab/lqab121 (PMC8759567; doi:10.1093/nargab/lqab121)
Supplement: lqab121_Supplemental_Files [file lqab121_supplemental_files.zip › Supplementary Figures_camera-ready-clean.docx]

**InterTADs: Integration of multi-omics data on topologically associated domains, Application to Chronic Lymphocytic Leukemia**

Supplementary Material


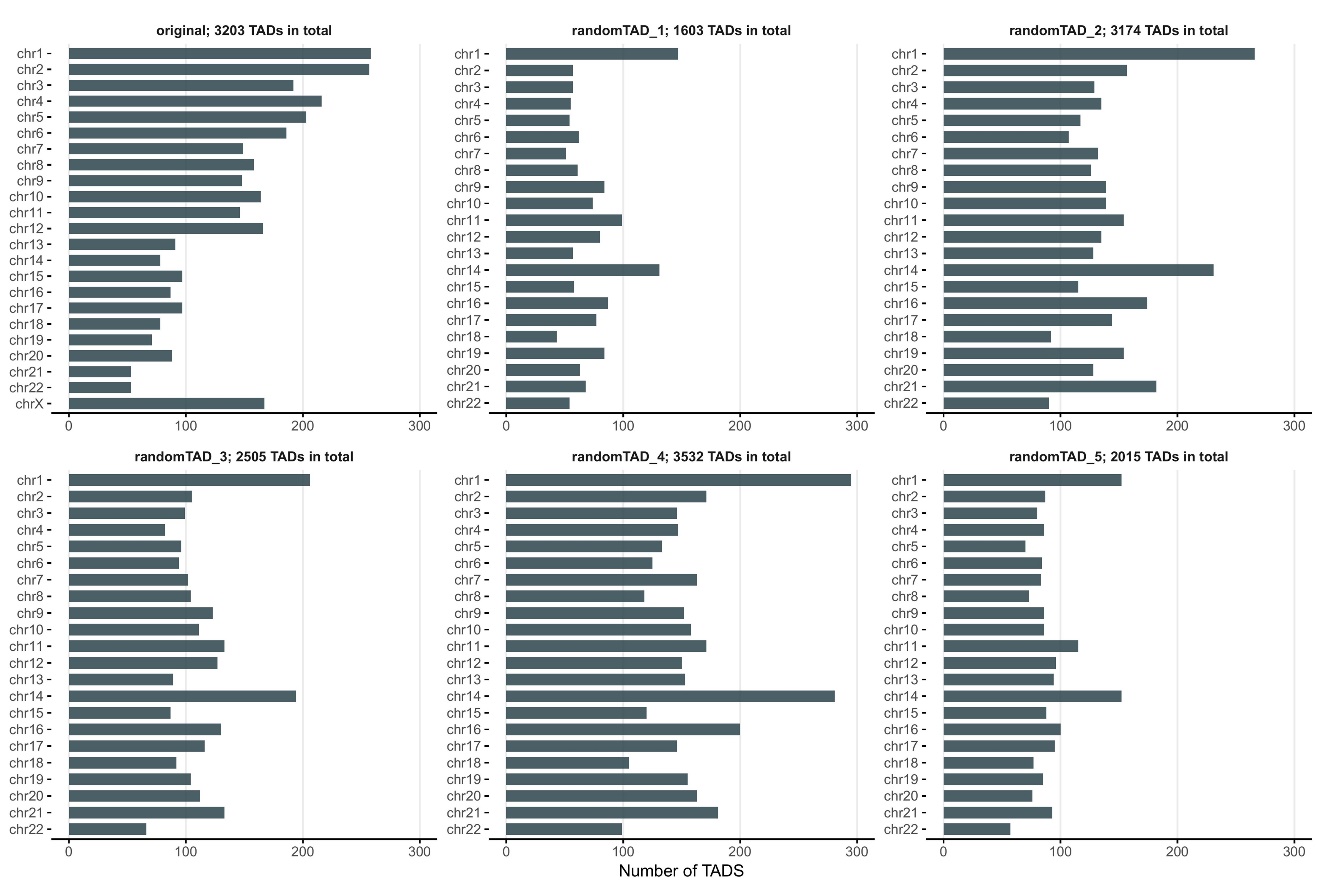


**Supplementary Figure 1:** Overview of the randomly generated TAD files. Bar plots depict the number of TADs for each chromosome. The first subgraph contains the number of TADs for the original TAD file, while the next subgraphs show the randomly generated TADs. The title of each subgraph shows the total number of TADs in each case.


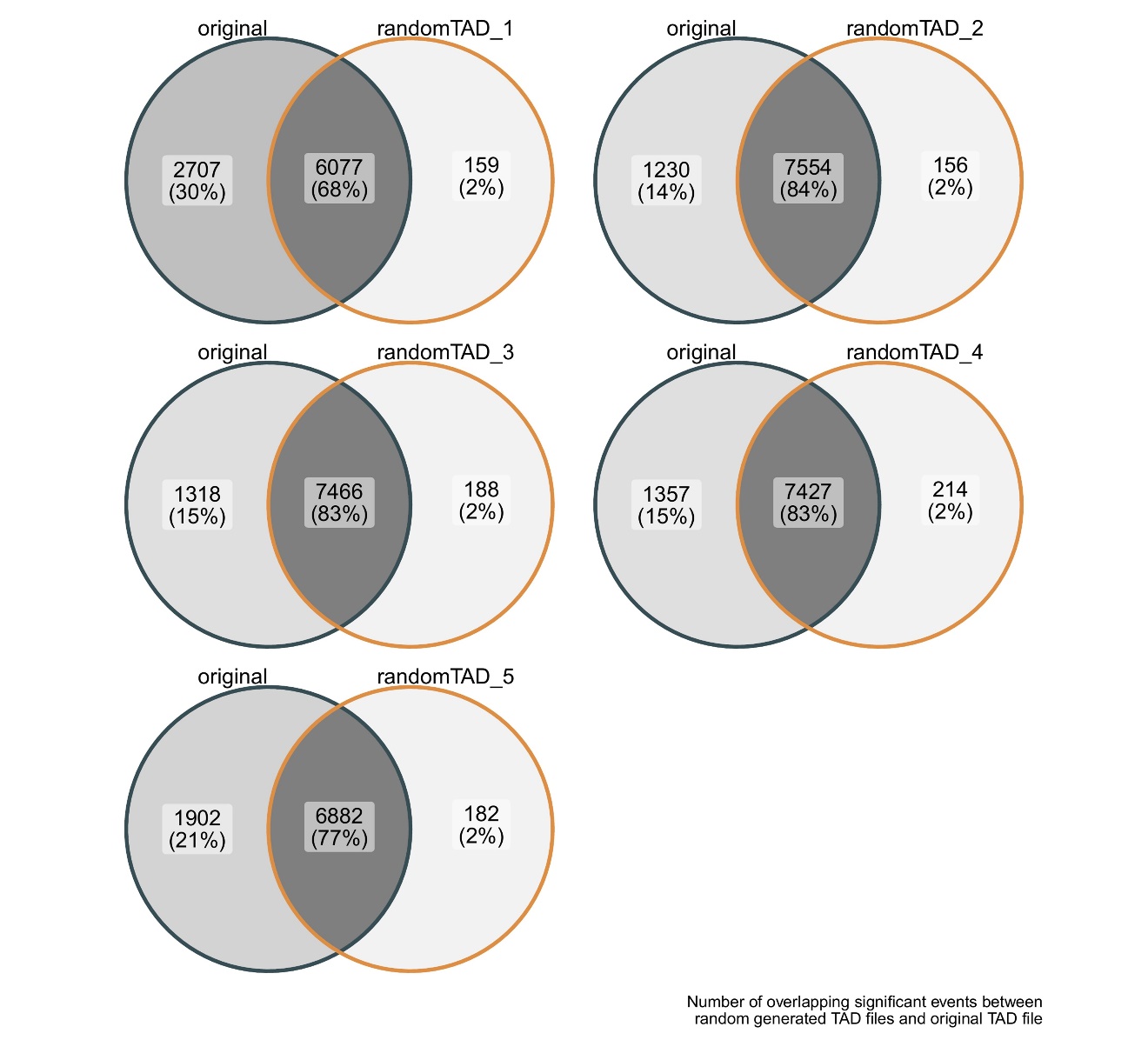


**Supplementary Figure 2:** Results assessment of the *evenDiff* module. The five Venn diagrams depict the overlap of the statistically significant events (CpGs, Genes) between the randomly generated TAD files and the original TADs.


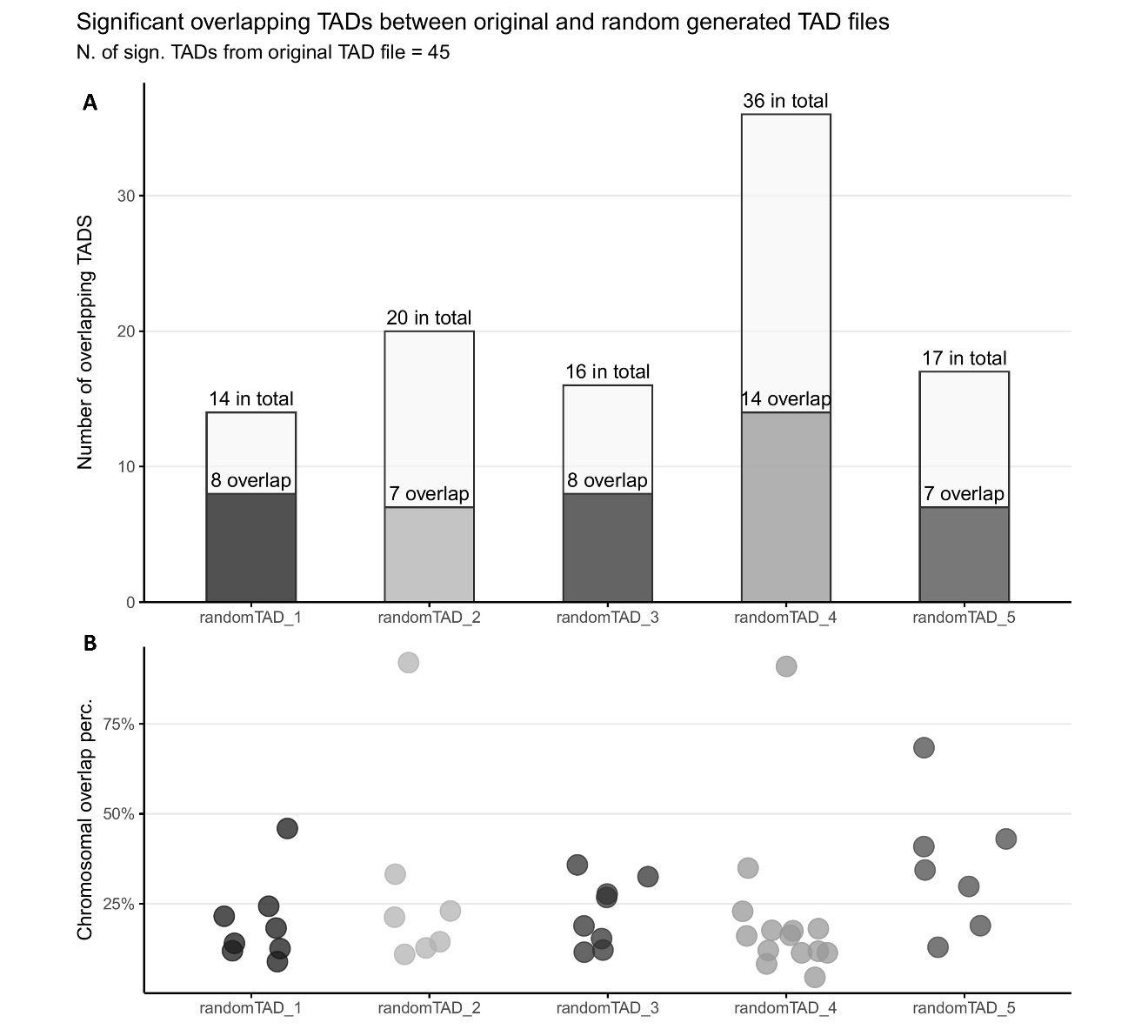


**Supplementary Figure 3:** Results assessment of the *TADiff* module. **(A)** The bar plots depict the number of significant TADs for each randomTAD file and the number of overlapping TADs (based on their chromosomal coordinates) with the original TAD file. **(B)** The dot plots correspond to the chromosomal overlap percentage of the overlapping TADs (randomly generated against the original).


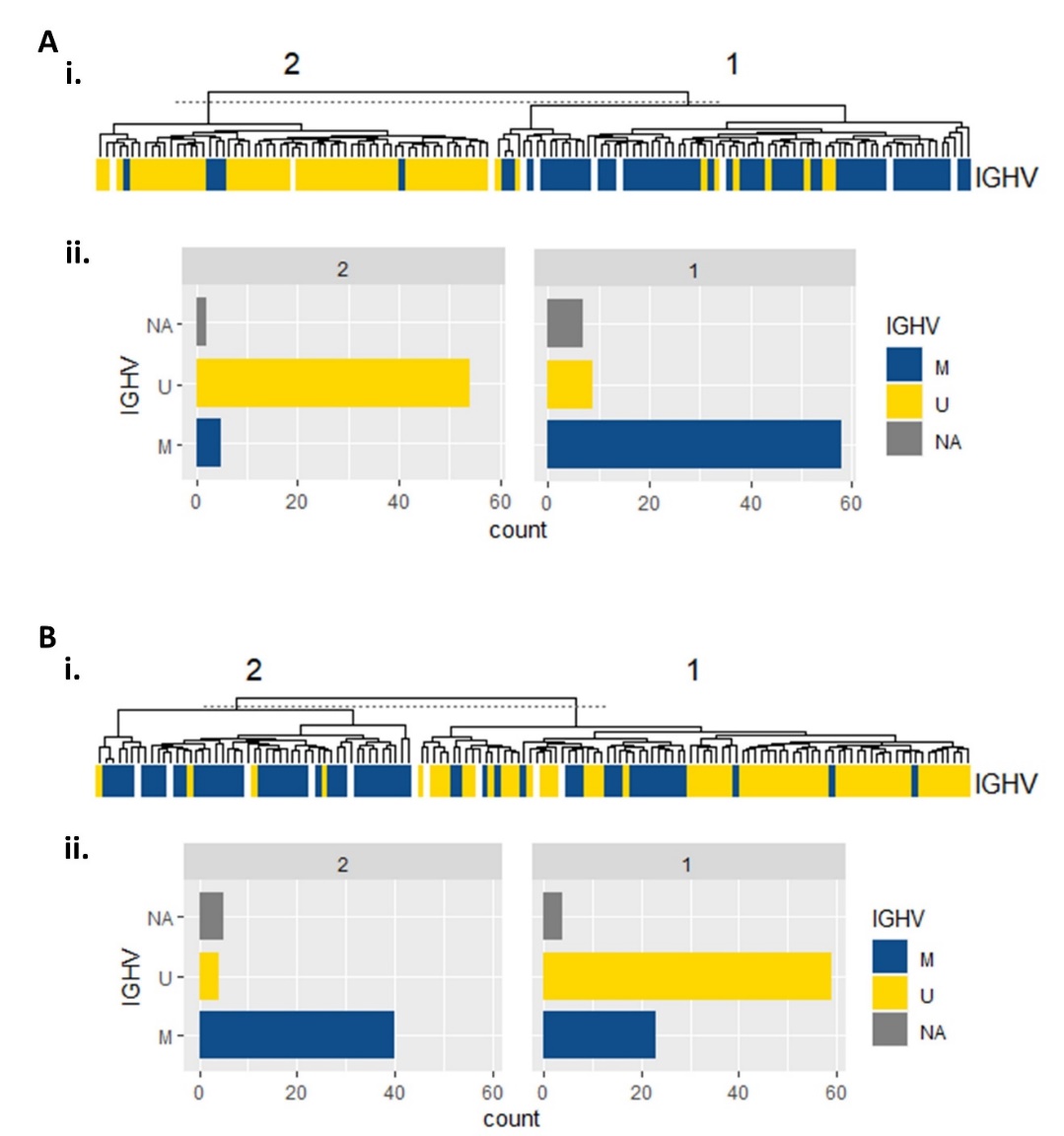


**Supplementary Figure 4.** Unsupervised hierarchical clustering analysis taking into account; **A.i** the CpGs and the transcripts, and **B.i** only the CpGs. The barplots (**A.ii** and **B.ii**) show the distribution of the M- and U- CLL cases in each cluster generated from the hierarchical clustering.

**
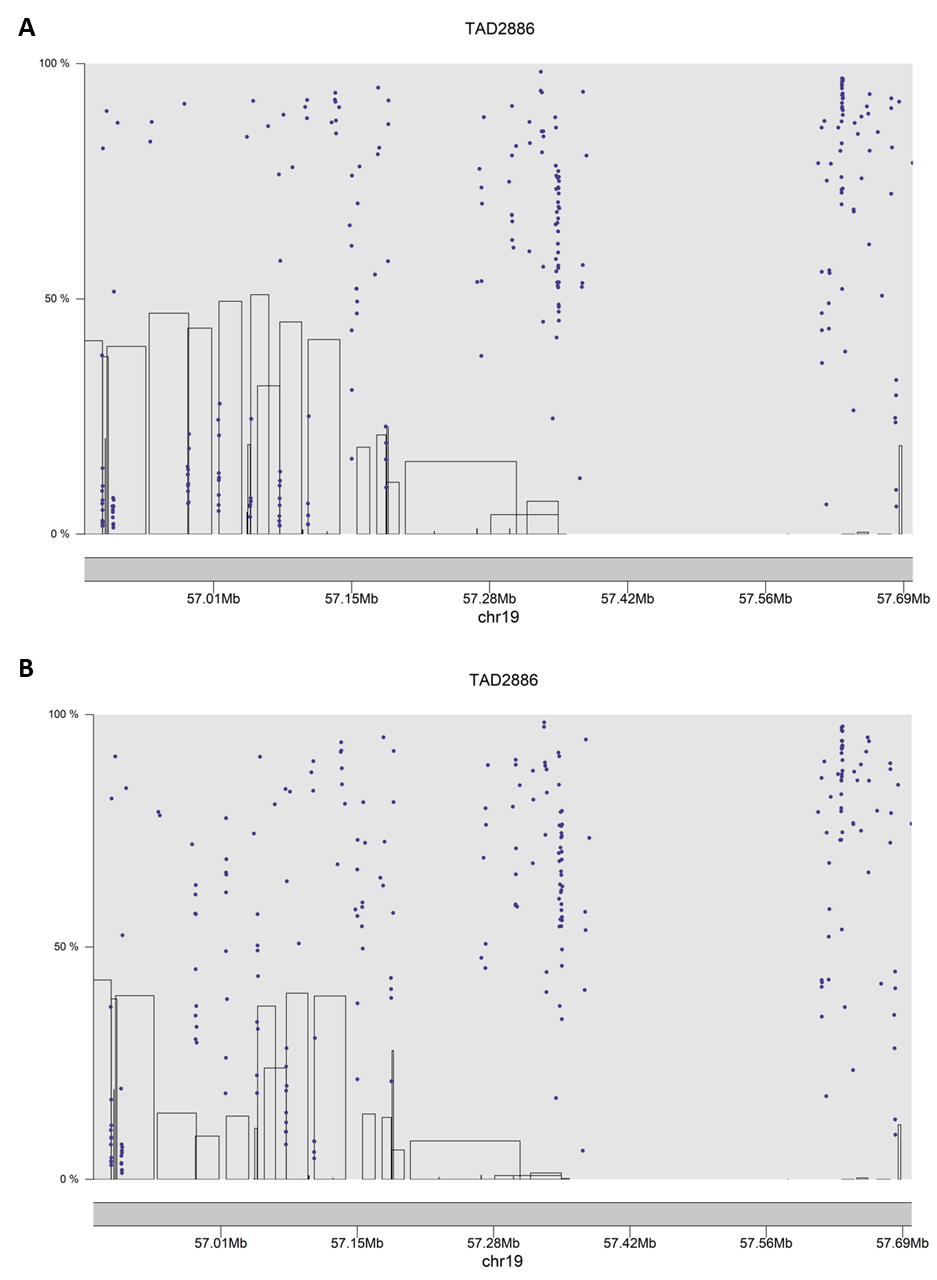
**

**Supplementary Figure 5.** Scatter plot showing the chromosomal region of the TAD2886 (x-axis) and the values of the events (y-axis) on **A.** U-CLL and **B.** on M-CLL. The black boxes state the presence of a transcript, the blue dot represents a CpG site.


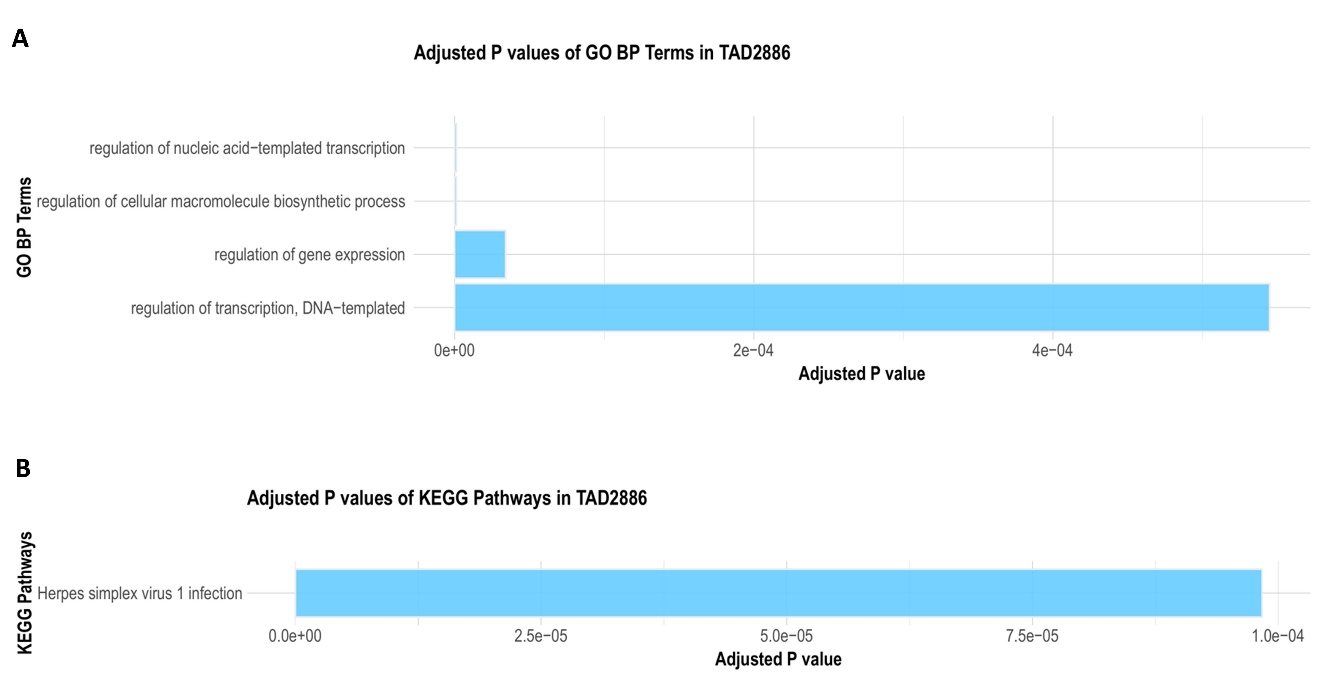


**Supplementary Figure 6.** Bar plot showing the significant **A.** GO terms and **B.** KEGG pathways based on the 37/160 events of TAD2886 on y-axis and the adj-pvalue of the enrichment analysis in x-axis


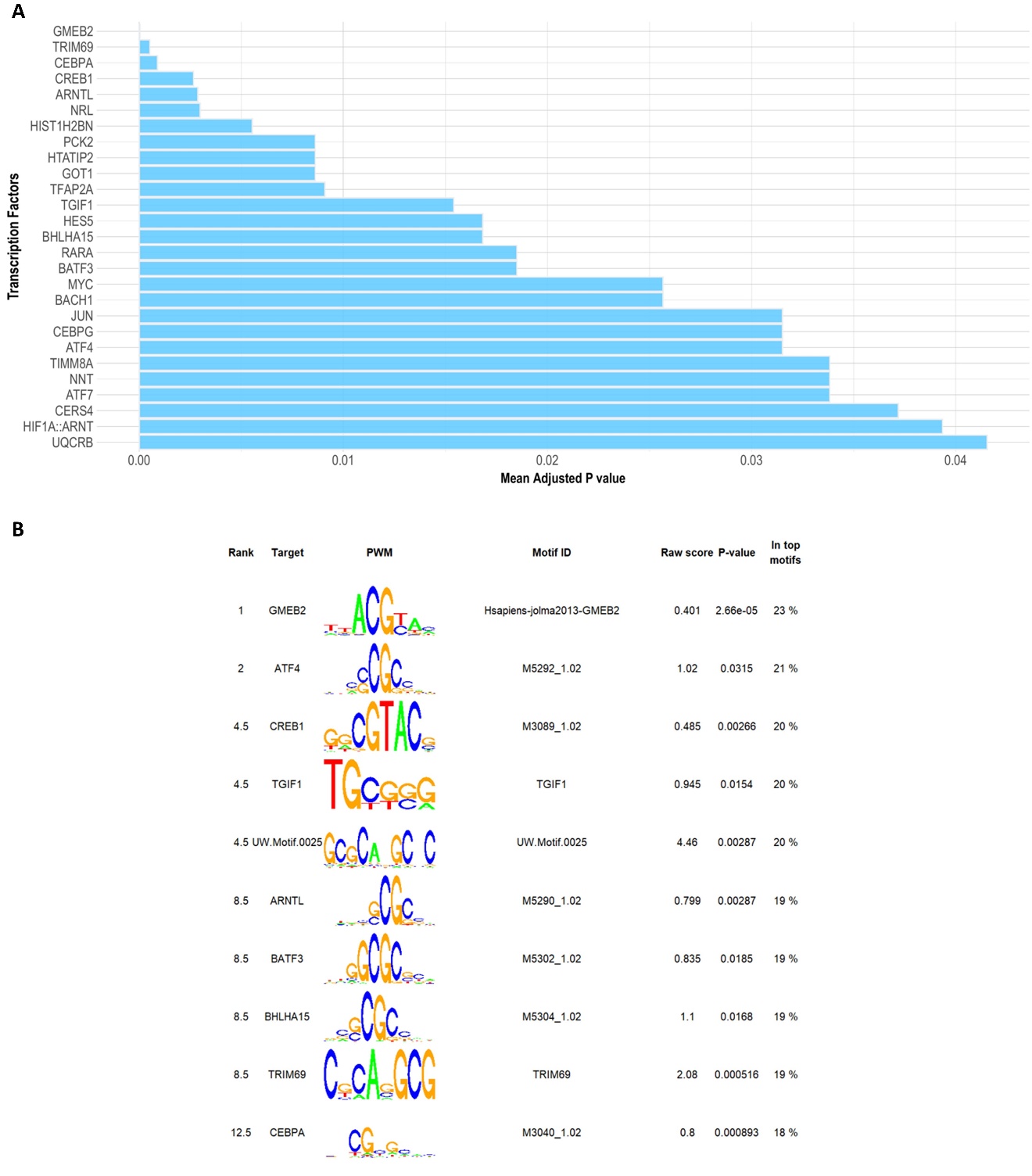


**Supplementary Figure 7. A.** Bar plot representing the statistically enriched TFs based on the 37/160 events of TAD2886 on y-axis and the mean adj-pvalue of the enrichment analysis in x-axis. **B.** Panel shows the motifs for each as well as the statistical analysis resulting from enrichment analysis
