# Supplementary material for: InterTADs: integration of multi-omics data on topologically associated domains, application to chronic lymphocytic leukemia
Source: NAR Genom Bioinform. 2022 Jan 14;4(1):lqab121. doi: 10.1093/nargab/lqab121 (PMC8759567; doi:10.1093/nargab/lqab121)
Supplement: lqab121_Supplemental_Files [file lqab121_supplemental_files.zip › Supplementary Table Legend.docx]

**Supplementary table captions**

**Supplementary Table 1:** Detected events through evenDiff module regarding the IG SHM status.

**Supplementary Table 2:** Detected events through evenDiff module regarding the presence of trisomy 12.

**Supplementary Table 3:** Detected TADs through TADiff module regarding the IG SHM status
